# Autochthonous Human Babesiosis Caused by *Babesia venatorum*, the Netherlands 

**DOI:** 10.3201/eid3009.240556

**Published:** 2024-09

**Authors:** Niekie Spoorenberg, Clara F. Köhler, Evelien Vermeulen, Suzanne Jurriaans, Marion Cornelissen, Kristina E.M. Persson, Iris van Doorn, Hein Sprong, Joppe W. Hovius, Rens Zonneveld

**Affiliations:** Amsterdam University Medical Center, University of Amsterdam, Amsterdam, the Netherlands (N. Spoorenberg, E. Vermeulen, S. Jurriaans, M. Cornelissen, I. van Doorn, J.W. Hovius, R. Zonneveld);; Amsterdam Institute for Immunology & Infectious Diseases, Amsterdam (N. Spoorenberg, S. Jurriaans, M. Cornelissen, J.W. Hovius, R. Zonneveld);; Centre for Infectious Disease Control, National Institute for Public Health and the Environment (RIVM), Bilthoven, the Netherlands (C.F. Köhler, H. Sprong);; Lund University, Lund, Sweden (K.E.M. Persson);; Skåne University Hospital, Lund (K.E.M. Persson)

**Keywords:** vector-borne infections, Babesia, ticks, *Babesia venatorum*, *Ixodes ricinus*, Netherlands, parasites

## Abstract

Severe babesiosis with 9.8% parasitemia was diagnosed in a patient in the Netherlands who had previously undergone splenectomy. We confirmed *Babesia venatorum* using PCR and sequencing. *B. venatorum* was also the most prevalent species in *Ixodes ricinus* ticks collected around the patient’s home. Our findings warrant awareness for severe babesiosis in similar patients.

Human babesiosis is a tickborne disease caused by protozoans that infect red blood cells. Immunocompromised persons and those who have undergone splenectomies are at risk for severe illness (*1*). Most confirmed cases of babesiosis in Europe have been in such patients and have most frequently been *Babesia divergens* infections (*1*). In the Netherlands, DNA of *Babesia* species have previously been reported in *Ixodes ricinus* ticks, blood of animals, and in several human samples (*2*–*4*). However, reports of autochthonous human babesiosis in the Netherlands are lacking. We report a case of autochthonous human babesiosis in the Netherlands, as well as data on *Babesia* DNA in ticks collected near the patient’s home. Handling of data and patient samples was performed according to the highest ethics standards. Informed consent was received from the patient.

## The Study

In September 2023, a 69-year-old man was admitted to the hospital with fever (38°C), malaise, macroscopic hematuria, and laboratory results indicative of acute hemolysis. His medical history reported Hodgkin disease stage 3A in 1977, which went into remission after splenectomy and mantle field radiotherapy, and a diffuse large B-cell lymphoma in 2015, also in remission after being treated with 6 cycles of chemotherapy treatment. Several months before this hospitalization, chronic inflammatory demyelinating polyneuropathy had been diagnosed, and he was treated with immunoglobulins, plasma exchange, and dexamethasone pulse therapy. 

**Figure 1:**
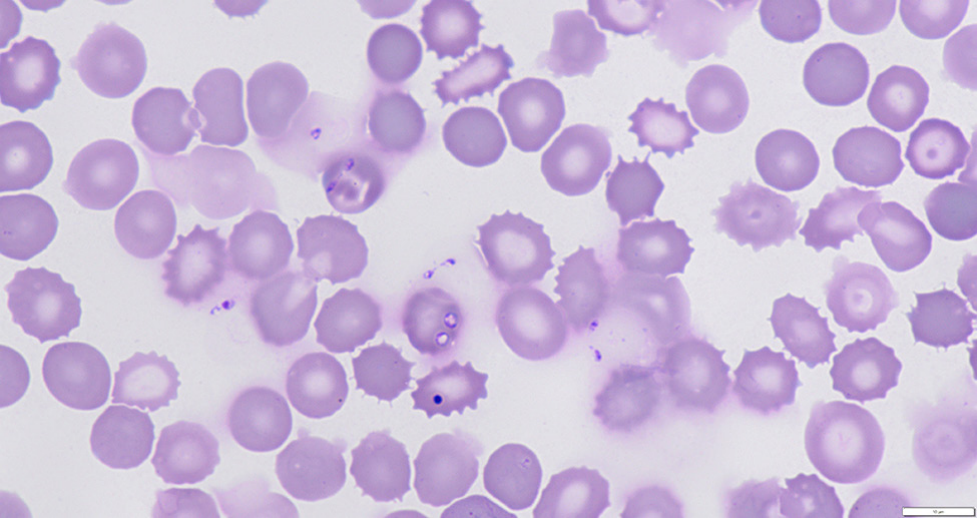
*Babesia venatorum* parasites in blood of patient with autochthonous human babesiosis, the Netherlands. Giemsa-stained thin smear from blood of patient before start of treatment and visualized with light microscopy (original magnification ×1,250) shows small and pleomorphic amoeboid and vacuolar asexual stages of *Babesia* spp., positioned within and outside red blood cells, which is typically observed in human babesiosis. Of note, 1 dark blue–stained Howell-Jolly body is observed.

**Figure 2:**
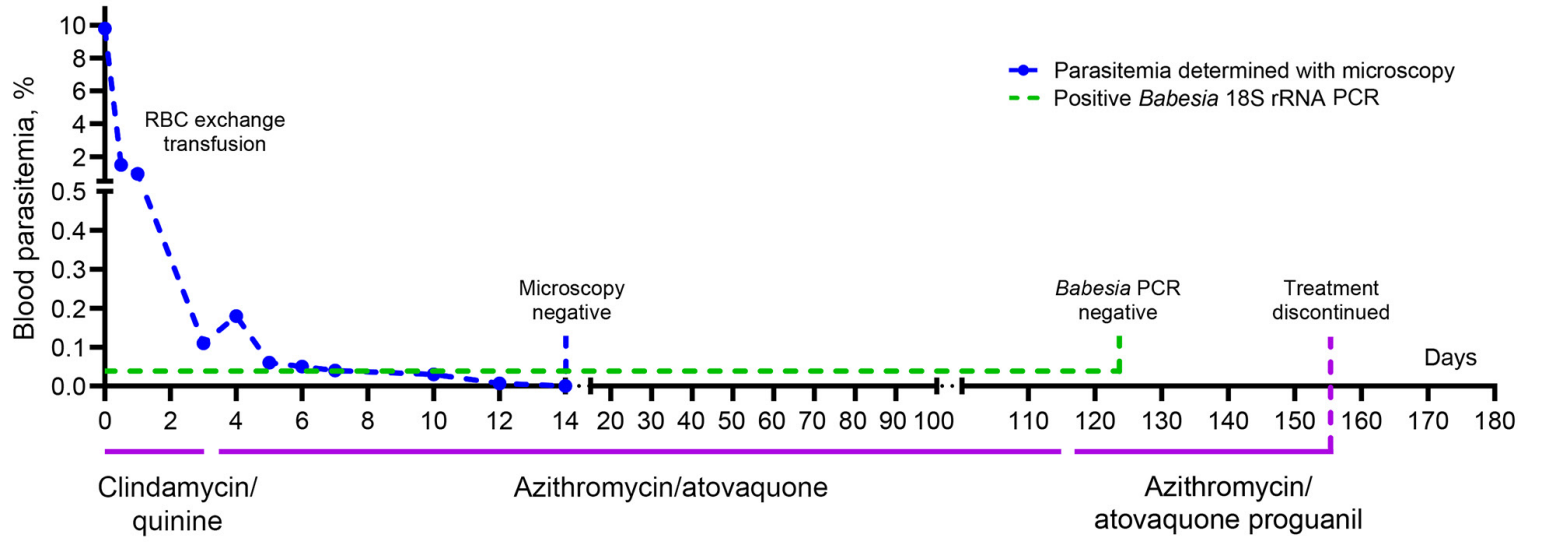
Clinical evolution of illness and treatment in case-patient with autochthonous human babesiosis caused by *Babesia venatorum*, the Netherlands. RBC, red blood cell.

At the hospital, microscopy on peripheral blood smears revealed red blood cell inclusion bodies. The patient did not report recent travel to malaria-endemic areas but recalled a tick bite that occurred 2 months before near his home. Additional microscopy showed parasites with morphologic features that resembled *Babesia* spp.; 9.8% of red blood cells were infected (Figure 1). Immediately after admission, a red blood cell exchange transfusion and intravenous treatment with clindamycin and quinine reduced parasitemia to <1%. Treatment was switched to oral azithromycin and atovaquone. After 14 days, parasites were no longer detectable with microscopy. *Babesia* DNA remained detectable despite treatment until 4 months after admission. After proguanil was added to the treatment regimen, *Babesia* DNA disappeared the next week. Oral treatment was discontinued approximately 6 weeks later. No relapse of babesiosis has occurred, despite reinitiation of immunosuppressive drugs (Figure 2). 

**Figure 3:**
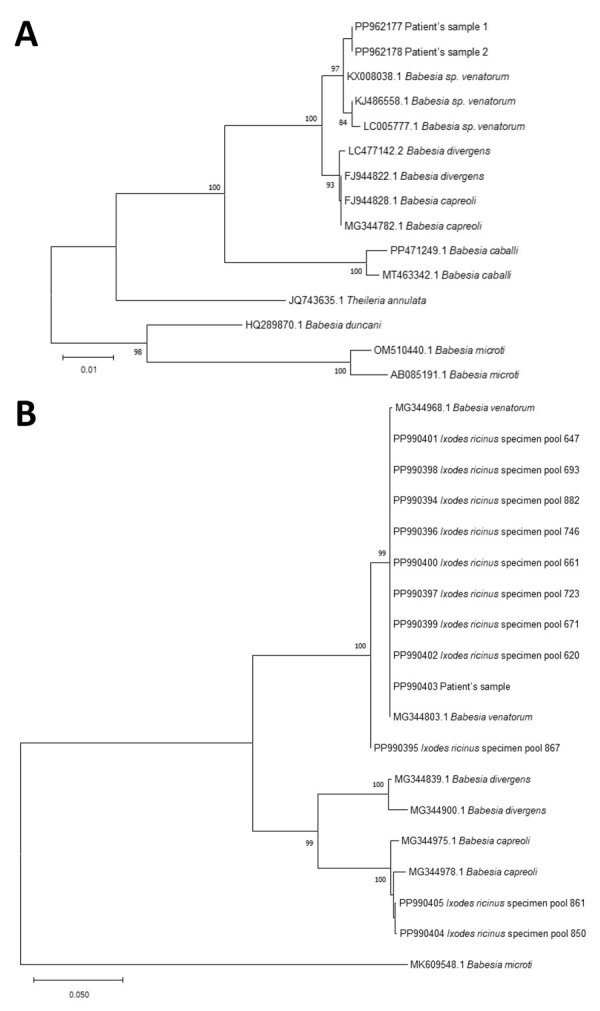
Phylogenetic analysis of *Babesia* sequences obtained from patient samples in study of autochthonous human babesiosis caused by *Babesia venatorum*, the Netherlands. A) Neighbor-joining tree of the phylogenetic relationship of *Babesia* species based on sequences of a fragment of 18S ribosomal RNA. B) *Babesia* sequences isolated from *Ixodes ricinus* ticks collected near the patient’s residence (*Ixodes ricinus* specimen pool 620, 647, 661, 671, 693, 723, 746, 850, 861, 867, and 882), of a fragment of cytochrome oxidase subunit 1. The distance between sequences was calculated using the Kimura 2-parameter model. The bootstrap test was performed with 500 replicates. Bootstrap values >70 are displayed. GenBank accession numbers are provided for all sequences.

We visualized parasites using fluorescence microscopy on acridine-stained blood in quantitative buffy coat capillaries and conventional microscopy on Giemsa-stained thick and thin smears (Figure 1). Results of histidine-rich protein 2 and aldolase antigen testing (Biozek, https://www.biozek.com) and malaria loop-mediated isothermal amplification (Meridian Bioscience, https://www.meridianbioscience.com) were negative. A *Plasmodium* 18S ribosomal DNA PCR with known cross-reactivity with *Babesia* spp. was positive (*5*). Sanger sequencing of the ≈125 bp PCR amplicon revealed 100% homology with both *B. divergens* and *B. venatorum*. *Babesia* 18S ribosomal RNA (628 bp) amplification and sequencing showed 100% homology with *B. venatorum* (Figure 3, panel A) but not with *B. divergens*. Mitochondrial cytochrome oxidase subunit I (COI) amplification and sequencing showed 100% homology with *B. venatorum* from animals and ticks in the Netherlands (*3*). Species-specific PCRs for 4 *Babesia* spp. further confirmed the presence of *B. venatorum* DNA. A crude merozoite extract–based *B. divergens* ELISA showed optical densities slightly above the cutoff (*6*). Low titers might have resulted from continued plasmapheresis or poor cross-reactivity of antibodies against *B. venatorum* with *B. divergens* antigens.

To assess current and local risk of acquiring babesiosis, we collected questing ticks in 3 areas within 2–3.5 km of the patient’s home using blanket dragging (*7*). We selected the areas on the basis of the presence of (wild) ungulates, which are amplifying hosts of *Babesia* sensu stricto (*3*). Roe deer were present in all areas. In addition, areas 1 and 2 were grazed by fallow deer (*Dama dama*), domestic cattle (*Bos taurus*), and Konik horses (*Equus caballus ferus*). Of note, in area 1, a small herd of European bison (*Bison bonasus*) was introduced in 2007. 

We collected a total of 2,786 *I. ricinus* ticks. Ticks were tested in pools because prevalence of *Babesia* DNA in ticks in the Netherlands is very low (*3*). If a pool was positive, only 1 tick was considered positive in that pool. Overall, 25 (0.9%) ticks were infected with *Babesia* s.s. based on amplification of mitochondrial COI (Table). From those *Babesia*-positive ticks, we amplified DNA with *Babesia* species–specific PCRs, which were positive for *B. venatorum* (n = 20), *B. capreoli* (n = 4), and the European variant of *B. odocoilei* (n = 1). Phylogenetic analysis demonstrated that the *B. venatorum* COI sequence from the patient clustered with *B. venatorum* sequences from ticks collected near the patient’s home (Figure 3, panel B). We calculated that 0.7% of ticks were infected with *B. venatorum*. The estimated incidence of tick bites in humans is 1.1 million/year, so human exposure to *B. venatorum* is ≈7,920 persons per year (*8*). 

**Table:** *Babesia* sensu stricto species found in nymphal and adult *Ixodes ricinus* ticks in study of autochthonous human babesiosis caused by *Babesia venatorum*, the Netherlands*

Area	No. ticks tested	No. positive results (% total collected)			
		*Babesia* s.s.	*B. capreoli*	*B. odocoilei*	*B. venatorum*
1	982	4 (0.5)	None	None	4 (0.4)
2	1,126	10 (0.9)	4 (0.4)	1 (0.1)	5 (0.4)
3	678	11 (1.6)	None	None	11 (1.6)
Total	2,786	25 (0.9)	4 (0.1)	1 (0.04)	20 (0.7)

*Ticks were collected in 3 natural areas near the patient’s residence. *Babesia* s.s. were identified to the species level by species-specific PCRs.

## Conclusions

This case of autochthonous human babesiosis concerned life-threatening disease in a highly susceptible patient who had a rare medical history of diffuse large B-cell lymphoma years after Hodgkin disease. He was presumably infected with *B. venatorum* after a tick bite. The clinical course was favorable after prompt therapy, and parasites were undetectable after 2 weeks. Persistent *Babesia* DNA disappeared after adding proguanil to the treatment regimen. Of all *Babesia* DNA found in local ticks, *B. venatorum* was most prevalent, indicating a higher chance of exposure to this species. This finding is notable because, in Europe, most described human babesiosis has been caused by *B. divergens*, much less frequently by *B. venatorum* or *B. microti* (*1*).

In the Netherlands, DNA of >8 *Babesia* spp. have been found in animals and ticks but not in humans (*2*–*4*). Positive serologic results in studies among blood donors and persons with increased tick bite exposure indicate that self-limiting and unnoticed infections might occur in the general population in Europe (*9*,*10*). However, further investigations in such populations did not report *Babesia* DNA as evidence for human babesiosis (*11*,*12*). Scarcity of cases might also be explained by lack of awareness and underdiagnosis or misdiagnosis. Indeed, in several published cases, diagnosis was delayed or made postmortem (*13*). The morphologic overlap between *Babesia* and *Plasmodium* spp. in microscopy is high, making sensitive and specific testing for malaria imperative to prevent misdiagnosis and associated treatment delays. Last, underdiagnosis might also be the result of the high proportion of human babesiosis that could occur after unnoticed bites from nymphal ticks.

Different *Babesia* spp. are specific to different animal reservoirs. Therefore, determining infecting *Babesia* spp. can help assign surveillance among ticks and specific animals. A previous study conducted in the Netherlands during 2000–2019 found a widespread occurrence of *B. venatorum* DNA in 0.8% of 25,849 sampled *I. ricinus* ticks (*3*). *B. venatorum* was present in 46% (n = 290) of sampled roe deer (*Capreolus capreolus*) and to a much lesser extent in sheep (*Ovis aries*). Roe deer are highly present in the areas investigated for this case report, and the patient had observed many around his home. Tick densities could have increased in recent decades and changes in wildlife management could have contributed to spread of *Babesia*-infected ticks (*14*). Those changes might have driven wildlife infected with *B. venatorum* into areas where the parasite was formerly absent. Unfortunately, we were not able to sample local ungulates to investigate *Babesia* spp. prevalence. Finally, climate change might affect spatial and temporal tick distribution and increase the risk for and prevalence of human babesiosis, which warrants further surveillance studies (*15*). Although it remains unclear which factors have driven this occurrence of human babesiosis, awareness among clinicians is warranted, especially for susceptible patients.
